# Test-Retest Reliability of Vibration Perception Threshold Test in People with Type 2 Diabetes Mellitus

**DOI:** 10.3390/ijerph17051773

**Published:** 2020-03-09

**Authors:** Francisco Javier Domínguez-Muñoz, José Carmelo Adsuar, Santos Villafaina, Miguel Angel García-Gordillo, Miguel Ángel Hernández-Mocholí, Daniel Collado-Mateo, Narcís Gusi

**Affiliations:** 1 Physical Activity and Quality of Life Research Group (AFYCAV), Faculty of Sport Science, University of Extremadura, 10003 Cáceres, Spain; fjdominguez@unex.es (F.J.D.-M.); svillafaina@unex.es (S.V.); mhmocholi@unex.es (M.Á.H.-M.); ngusi@unex.es (N.G.); 2 Health Economy Motricity and Education (HEME), Faculty of Sport Science, University of Extremadura, 10003 Cáceres, Spain; jadssal@unex.es; 3 Facultad de Administración y Negocios, Universidad Autónoma de Chile, Sede Talca 3467987, Chile; 4 Centre for Sport Studies, Rey Juan Carlos University, Fuenlabrada, 28943 Madrid, Spain; danicolladom@gmail.com

**Keywords:** type 2 diabetes mellitus, reliability, Vibratron II, vibration perception threshold

## Abstract

Background: Diabetes mellitus is a chronic disease characterized by fasting hyperglycemia. It affects approximately 415 million people worldwide and involves a variety of complications. One of them is the loss of sensitivity to peripheral vibration. Objective: Our study aims to discover the test-retest reliability of a procedure for assessing vibration sensitivity in people with type 2 diabetes mellitus. Methodology: 90 people with type 2 diabetes mellitus (56 men and 34 women) performed the vibration perception threshold (VPT) test using the Vibratron II device. A re-test was completed seven days after the first reading. Results: The relative reliability of the VPT test result is excellent (intraclass correlation coefficient = 0.96). The same applies to gender and obesity subgroups. Regarding absolute reliability, the standard error of measurement is 8.99%, and the small real difference is 24.94%. Conclusions: The relative and absolute reliability results of the vibration perception threshold in people with type 2 diabetes mellitus offer excellent results.

## 1. Introduction

Diabetes Mellitus (DM) is “a general term for heterogeneous disturbances of metabolism for which the main finding is chronic hyperglycaemia. The cause is either impaired insulin secretion or impaired insulin action or both” [1]. This disease affects approximately 415 million people worldwide aged 20–79, confirming that it is one of the most significant health problems in our society. Furthermore, according to the International Diabetes Federation (IDF), about 46.5% of people suffering from DM have not been diagnosed yet, which may change the prevalence data of this disease. An estimated 642 million people will be diagnosed with DM worldwide in 20 years [2]. It affects a significant percentage of the world’s population and carries a series of secondary consequences, such as high health costs, productivity loss, and disability [3]. Accordingly, DM can produce a set of complications, such as cardiovascular problems, retinopathy, and nephropathy. Another complication is the loss of sensation in peripheral parts of the body, known as peripheral neuropathy, which affects up to 50% of patients with DM [4]. 

Diabetic peripheral neuropathy is characterized by a progressive loss of sensitivity in the farthest parts of the body, eventually affecting small-diameter nociceptive skin fibers [5]. It can also alter the motor fibers, leading to a series of complications such as muscle weakness. People with DM suffer from a strength reduction of 17% and 14% on the knee’s flexor and extensor muscles, respectively [6]. It is essential to consider these aspects since this type of problem can affect balance, leading to alterations in posture and gait [7]. Several studies support these balance problems theories in people with type 2 DM, including ankle and foot proprioception deficits [8], or a loss of sensation in feet [9].

Several tests used for the diagnosis of diabetic peripheral neuropathy are associated with tests conducted on the lower extremities [10]. If there is such a perception deficit in the extremities, it can lead to more critical problems such as ulcers [11]. 

Quantitative sensory testing (QST) is a non-invasive method for measuring and quantifying nerve function [12]. There are multiple tests for this type of analysis, and one of them is the Vibratron II, an affordable and accessible tool for measuring vibration sensibility. This tool has also been used to test reliability in people with other illnesses, such as low back pain [13]; however, to our knowledge, it has not yet been used in people with type 2 DM.

The vibration perception threshold (VPT) is a measure considered as a valid indicator of proprioceptive capacity [14]. There are some reliability studies of different tests related to proprioception and balance in patients with type 2 DM [15]; however, a systematic search recently conducted indicates that there is a lack of such studies for the DM population [16].

Therefore, our study aims to determine the test-retest reliability of a procedure for assessing vibration sensitivity (aspect related to proprioception and balance) in people with type 2 DM. 

## 2. Materials and Methods 

### 2.1. Sample Calculation

The sample size was calculated to achieve a 0.90 potential for an intraclass correlation coefficient (ICC) under the following assumption: alpha = 0.05. The null hypothesis stated that the ICC was “good” based on the criteria used (0.70) [17]. The alternative hypothesis stated that the ICC was excellent (0.90) according to the same criteria. A minimum of 25 participants was required for each test session. For all the subgroup analyses conducted, there was a minimum of 25 participants, ensuring an adequate sample calculation.

### 2.2. Participants

A total of 90 people with type 2 DM participated in the study, 56 men and 34 women. The following inclusion criteria were mandatory to participate in the study: (a) men or women with type 2 DM diagnosis between 40 and 85 years old, and (b) have read, accepted, and signed the written informed consent. The following exclusion criteria were used for the study: (a) have a condition that may make the high intensity exercises contraindicated, such as retinopathy, musculoskeletal injuries, major balance problems, or high risk of thrombosis, (b) be under psychotropic or neurotoxic treatment, (c) be exposed to neurotoxins (industrial accidents or be in contact with toxic residues), (d) receive radiation therapy, (e) high risk of non-diabetic neuropathy (such as HIV, alcoholism, or uremia), (f) have or had a job with high exposure to mechanical whole body vibrations, and (g) have performed whole body vibration exercises prior to this intervention. This study was approved by the Bioethics Committee of the Universidad de Extremadura (44/2012) and conducted in accordance with the Helsinki Declaration, as well as national legislation on bioethics, biomedical research, and confidentiality of personal data.

### 2.3. Procedure

All participants were informed of the study objectives and agreed to sign the informed consent, as indicated above. Afterward, they were asked to provide socio-demographic data such as age, years of diagnosis, and number of falls in the last six months. After the interview, a body composition analysis was done. This analysis was performed by the Tanita BC-418 MA bioimpedance machine, which provided anthropometric values.

Then, the VPT was assessed using the Vibratron II^®^ (Physitemp Instruments, Inc: Clifton, NJ, USA), according to the supplier’s instructions. Hereunder was the procedure followed and the characteristics of the device:

### 2.4. Vibratron II ^®^

The equipment is made of a vibration control device and two vibratory modules. The controller device has a screen that displays the vibration amplitude, a vibration controller, and four switches; two to connect the equipment and adjust the amplitude, and another two to send the vibration amplitude from one module to another. One of the two switches sends it, and the other one is “fake” and only makes noise; however, it does not change the amplitude of the module vibration. With this configuration, the subject won’t be able to associate the sound with the vibration when switching from one module to another.

The vibratory modules dimensions are 12.5 × 8.5 × 23.5 cm, and each has a mark, A or B. Besides, each module has a 9.5 cm high with a 1.5 cm diameter cylindrical pivot to support the big toe. There is also a pair of padded mats to place the modules to avoid transmitting the vibration through the floor. These modules vibrate at a 120 Hz frequency, and the vibration amplitude is measured in vibration units. These vibration units relate to the movement amplitude in microns and according to the following formula:

A = x^2^/2 (1)

where x is the vibration units (vu), and A is the amplitude expressed in microns (μ).

### 2.5. Sensitivity Recording 

The protocol used in this study was one of the two proposed by the device manufacturer, specifically the one called “Two-alternative forced choice procedure”. For this procedure, the subject was asked to place the fingertips on the cylindrical pivots (the second finger for the upper limbs and the first toe for the lower limbs). Our study examined the lower limbs. Once the fingers were placed, the controller was connected; then, a sequence started whereby the vibrating elements were alternated manually by the research technician according to a table of elements labeled “A” and “B,” along with the vibration amplitudes to set in the device. This sequence started at high amplitude perceptible by the person; then, it was lowered and recorded when the individual answered correctly. This happened when their choice coincided with the vibrating module. Once the person made a mistake or could not recognize which module was vibrating, the vibration amplitude increased. This bi-directional process continued until the subject made five mistakes. The equipment’s manual suggested an increase and decrease range for the vibration of 5–10%.

### 2.6. Setting the Threshold 

Once the test was completed, the VPT was determined, following the manufacturer’s instructions described by Deng et al. [18]. The researcher was required to calculate the average value out of eight to state the VPT (from the last five hits excluding the lowest one, and the five mistakes, excluding the highest one). The proposal for this calculation form is called the alpha-cut mean (for this case, 20% cut), and it shows the authors’ concern about the fact that data may contain extreme random-generated values that could influence the vibration threshold. Two measurements were performed (seven days apart) to obtain the VPT. 

### 2.7. Statistical Analysis

The statistical software NCSSTMTM Pass v.11 software (NCSS, LLC. Kaysville, UT, USA) was used to measure sample size. Descriptive data were presented as means and standard deviations, and the statistical package SPSS 21.0 was used for their calculation. The ICC was used to determine the relative reliability, with the 95% confidence interval result in addition to the descriptive data. The SPSS 21.0 statistical package estimated the ICC (2,1). ICC estimates and their 95% confident intervals were calculated using SPSS statistical package version 21 (SPSS Inc, Chicago, IL, USA) based on: two-way random effects, absolute agreement, single rater. Munro’s rating was used for interpreting the ICC, ranging from 0.50 to 0.69 as moderate, 0.70 to 0.89 as good, and above 0.90 as excellent [17]. The standard error measurement (SEM) and the small real difference (SRD) were used to calculate absolute reliability. Absolute indexes were measured according to the expression:

(2) $$SEM=TD\;\sqrt{1-CCI}$$

where TD is the typical deviation of the data set. 

The SRD was determined according to the expression [19]:

(3) $$SRD=1.96\;\times SEM\;\times \;\sqrt{2}$$

as well as in percentages regarding the global measure average [20]. 

All these data were calculated with the Microsoft Office^TM^ Excel v.16 program. On the other hand, a step-by-step regression analysis was performed to explain the threshold through age and height. This analysis was executed with the SPSS 21.0 statistical package.

Additionally, the Bland-Altman [21] graphs were provided for the different estimators used to calculate the VPT. The analysis significance was previously established at *p* < 0.05. Graphics were made by GraphPad Prism 8 software. 

## 3. Results

Table 1 shows the sample’s essential characteristics (*n* = 90). The table was divided by gender, 56 men and 34 women, as well as by people with or without obesity. Participants with a body mass index (BMI) equal to or above 30 kg/m^2^ were considered obese, while those with a BMI below 30 kg/m^2^ were considered non-obese. The table shows age, anthropometric values, glycosylated hemoglobin, number of years with diagnosed diabetes, and number of falls in the last six months for the past year.

Table 2 shows the means and standard deviations of both measurements, with seven days in between. It also includes the *p*, which indicates the statistically significant differences between both measures. It can be observed that these differences exist for both measurements, for the entire sample in women and the non-obese population.

Table 3 contains the relative and absolute reliability of the VPT through ICC, SEM, and SRD. It shows excellent relative reliability, according to Munro et al. [17], since all groups’ results were above 0.90.

Table 4 shows the linear regression with the VPT relationship regarding height and age. The results show a model with an R^2^ of 0.362 and statistically significant differences in the association of VPT with height and age. 

**Figure 1:**
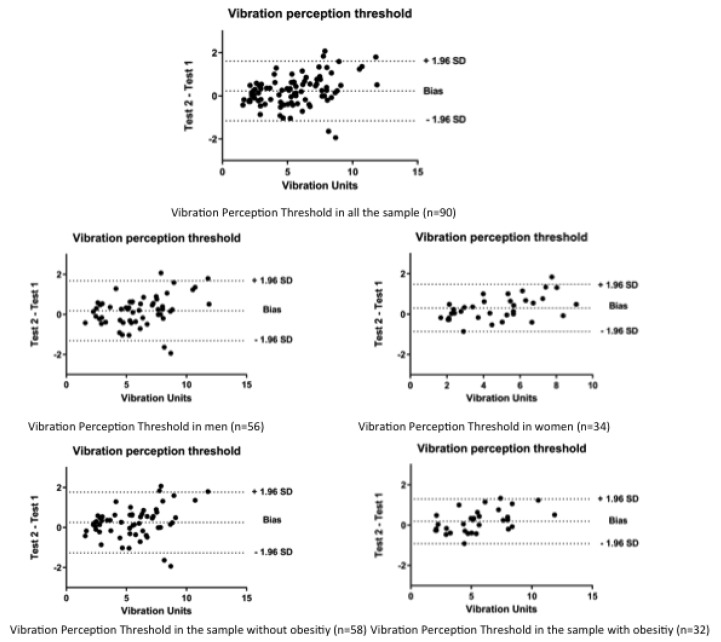
The Bland–Altman graphs of all groups analyzed in the study.

According to Figure 1, we can see the Bland–Altman graphs of all groups analyzed in the study.

## 4. Discussion

The main finding of this study is the excellent results of the relative test-retest reliability of the VPT values, as they were all above 0.90 [17] for both the whole sample (0.96) and the gender subgroups. The values were 0.95 for men, 0.96 for women, 0.97 for the obese, and 0.95 for the non-obese subgroups.

Other studies address the reliability of the vibrotactile threshold through different devices [22,23] and with other populations [13]. However, as far as we are aware, this is the first study researching the VPT reliability in type 2 DM by gender segregation.

This study would become the first one reporting SRD values from the VPT measurement test in people with type 2 DM. Based on the percentages obtained for SRD values, a general guideline can be set for patients with this disease: an observed change of greater than 24.94% is not due to within person variability in the measure based on repeated measures performed on the same participant a short time apart.

These results should be considered by researchers and clinicians whenever they use this type of test in that kind of population. The Vibratron II test is considered one of the tools included in the sensory quantification tests [12]. It is appropriate for the clinical diagnosis of different pathologies, such as carpal tunnel syndrome or diabetic neuropathy.

This VPT estimate can be considered as an indicator of diabetic neuropathy, which may influence their health-related quality of life, as reported in a study involving patients with and without this illness [24]. Suffering from neuropathy also leads to balance problems and mobility disorders [25,26], and increases the risk of falls [27]. The problem with being at risk of falling are bone fractures [28,29,30].

The VPT’s reliability has been investigated since its beginning [31,32,33,34]. Recently, studies using the ICC have been published assessing the reliability of the big toe in a healthy population [35], finding excellent values for both the right foot (ICC = 0.75) and the left one (ICC = 0.99). Reliability has also been found in the elderly [36] with an ICC of 0.89, as well as in people with diabetic neuropathy [23] with an ICC of 0.95 for all subjects (0.78 for the healthy ones and 0.94 for those with the disease). For the Vibratron II, only two reliability studies were carried out. These studies were conducted with healthy populations [37] and people with low back pain [13]. Therefore, this is the first study obtaining excellent absolute and relative reliability results, segregated by sex and obesity, in patients with type 2 DM, which will serve researchers and clinicians to use the values as a reference for future studies with VPT in patients with type 2 DM.

In this study, it is determined that height and age influence VPT in people with type 2 DM (Table 4). These results are consistent with previous studies [38,39,40,41]. It is known that age is the main determinant of VPT values, increasing VPT with age [22,36,42]. On the other hand, height influences the speed of motor nerve conduction [43,44], as these nerves are longer, so people with greater height have a greater probability of having worse VPT [45,46,47].

Previous studies have shown that BMI influences VPT in healthy people [48]. This theory may be because the Pacini and Meissner mechanoreceptors are located in the dermis, and the adipose tissue layer can reduce the VPT. However, in people with DM, this is somewhat controversial. Some studies indicate that BMI does influence VPT values [38], but some do not [49]. Data obtained in this article cannot confirm whether this relationship applies to people with type 2 DM. 

On a different note, in our results, there is a moderate direct correlation (Spearman’s Rho = 0.237; *p* = 0.025) between HbA1c and the VPT. These results follow the same line as previous studies conducted in people with type 2 DM [50,51]. A moderate direct correlation (Spearman’s Rho = 0.228; *p* = 0.031) has also been found between years of diagnosis and the VPT. It has been previously described in the literature that there is a deterioration of TPV depending on the years of diagnosis [52].

The current study has some limitations that should be taken into account. One of the limitations is worth noting that there is variability among devices in terms of the indicated voltage and the actual stimulator movement [23]. This fact suggests that there may be a lack of comparability among results from different biothensiometers. Thus, to avoid variability among devices and researchers, the VPT measurements were performed by the same equipment and evaluator. Data were expressed as percentages to compare the absolute reliability of the different VPT measuring devices. 

## 5. Conclusions

The relative reliability results of the vibration perception threshold test in people with type 2 diabetes mellitus is excellent, both for the general group and for the sex and obesity subgroups. As for the small real difference, the results for all cases were below 30%.

## Figures and Tables

**Table 1:** Baseline characteristics of the study participants.

	All (*n* = 90)	Men (*n* = 56)	Women (*n* = 34)	Without Obesity (*n* = 58)	With Obesity (*n* = 32)
Age (years)	65.64 ± 8.65	65.51 ± 8.16	65.85 ± 9.52	66.01 ± 8.16	64.96 ± 9.57
Height (cm)	164.89 ± 10.00	169.93 ± 7.80	156.59 ± 7.37	165.76 ± 9.50	163.31 ± 10.82
Weight (kg) *	80.63 ± 16.19	85.41 ± 17.28	72.74 ± 10.29	74.74 ± 10.47	91.29 ± 19.20
Fat Mass (%) *	32.97 ± 7.49	28.89 ± 5.20	39.68 ± 5.62	30.67 ± 6.50	37.12 ± 7.47
Fat Free Mass (%) *	66.84 ± 8.08	71.11 ± 5.21	59.80 ± 7.00	69.33 ± 6.49	62.32 ± 8.79
Body Water (%) *	49.08 ± 5.47	52.05 ± 3.81	44.20 ± 4.11	50.77 ± 4.73	46.03 ± 5.46
Basal Metabolism (kcal) *	161.61 ± 482.97	1818.70 ± 524.72	1323.47 ± 113.90	1528.02 ± 260.39	1819.37 ± 698.95
BMI (kg/m^2^) *	29.65 ± 4.39	29.63 ± 4.77	29.67 ± 3.74	27.10 ± 1.94	34.26 ± 3.80
Glycosylated Hemoglobin (%)	6.78 ± 1.02	6.85 ± 0.98	6.67 ± 1.09	6.79 ± 1.13	6.77 ± 0.80
Years of diagnosis	9.96 ± 8.83	9.55 ± 7.49	10.62 ± 10.78	9.26 ± 7.64	11.22 ± 10.68
Falls in 6 months	0.25 ± 0.71	0.12 ± 0.50	0.47 ± 0.92	0.31 ± 0.84	0.15 ± 0.36
Falls in 1 year	0.46 ± 1.21	0.17 ± 0.74	0.94 ± 1.65	0.53 ± 1.42	0.34 ± 0.70

BMI: body mass index; m: meters; * These values were obtained through the Tanita BC-418 MA bioimpedance machine.

**Table 2:** Vibration units in the Vibratron 2.0 test to measure the vibration perception threshold in 2 measurements with an interval of 7 d.

		Day 1 Mean ± TD	Day 2 Mean ± TD	*p* *
All (*n* = 90)	Vibration Threshold (vu)	5.43 ± 2.32	5.66 ± 2.55	0.003
Men (*n* = 56)	Vibration Threshold (vu)	5.93 ± 2.37	6.11 ± 2.61	0.076
Women (*n* = 34)	Vibration Threshold (vu)	4.59 ± 2.00	4.90 ± 2.30	0.006
Without obesity (*n* = 58)	Vibration Threshold (vu)	5.44 ± 2.31	5.69 ± 2.51	0.017
With obesity (*n* = 32)	Vibration Threshold (vu)	5.40 ± 2.38	5.59 ± 2.66	0.064

vu: vibration units; TD: typical deviation; * *p* values were calculated through paired samples test.

**Table 3:** Test-retest reliability of the vibration perception threshold test in 2 measurements with an interval of days between measurements.

		ICC (95% CI)	SEM (Nvu)	SEM (%)	SRD (Nvu)	SRD (%)
All (*n* = 90)	Vibration threshold (vu)	0.958 (0.938, 0.972)	0.49	8.99	1.38	24.94
Men (*n* = 56)	Vibration threshold (vu)	0.953 (0.922, 0.972)	0.53	8.96	1.49	24.85
Women (*n* = 34)	Vibration threshold (vu)	0.962 (0.924, 0.981)	0.41	8.83	1.16	24.48
Without obesity (*n* = 58)	Vibration threshold (vu)	0.949 (0.915, 0.969)	0.54	9.77	1.50	27.10
With obesity (*n* = 32)	Vibration threshold (vu)	0.975 (0.949, 0.988)	0.39	7.25	1.10	20.09

Vu: vibration units; ICC: intraclass correlation coefficient; CI: confidence interval; SEM: standard error measurement SRD: small real difference.

**Table 4:** Linear regression model explaining root vibration threshold (*n* = 90).

Variables	Vibration Perception Threshold (R^2^ = 0.362)		
	β	SE	*p*
Age	0.163	0.026	<0.001
Height	0.108	0.023	<0.001

SE: Standard Error.
